# Safety and efficacy of PCSK9 inhibitors and effect on coronary plaque phenotype in statin-treated patients following acute coronary syndrome: a systematic review and meta-analysis

**DOI:** 10.1186/s43044-024-00567-2

**Published:** 2024-10-04

**Authors:** Dennis Ievan Hakim, Faqrizal Ria Qhabibi, Muhammad Yusuf, Nasim Amar, Indra Prasetya, Ade Meidian Ambari

**Affiliations:** 1 Medical School Department, Faculty of Medicine, Brawijaya University, Jl. Veteran, Lowokwaru, Malang, 65145 Indonesia; 2 Department of Cardiovascular Prevention and Rehabilitation, National Cardiovascular Center Harapan Kita, West Jakarta, Jakarta Indonesia; 3 Department of Cardiology and Vascular Medicine, Faculty of Medicine, Brawijaya University– Dr. Saiful Anwar Hospital, Malang, Indonesia; 4 Department of Cardiology and Vascular Medicine, Faculty of Medicine, Universitas Indonesia – National Cardiovascular Center Harapan Kita, Jakarta, Indonesia

**Keywords:** Acute coronary syndrome, Atherosclerosis, LDL-C, Proprotein convertase subtilisin/kexin type 9, Statin

## Abstract

**Background:**

Acute coronary syndrome continues to be a significant cardiovascular issue. Statins are commonly acknowledged as medications that reduce LDL-C levels and stabilize plaques. Nevertheless, their efficacy is limited. Presently, PCSK9 inhibitors are suggested to be advantageous in patients who are already receiving statin treatment. The study seeks to assess the safety and effectiveness of PCSK9 inhibitors in individuals who have been treated with statins after experiencing acute coronary syndrome (ACS), as well as investigate the impact on the characteristics of coronary plaque.

**Methods:**

Articles were identified from PubMed, Cochrane Central Register of Controlled Trials, and ProQuest. Our analysis comprised trials and observational studies that compared the plaque phenotype, lipid profile, and safety outcomes between PCSK9 inhibitors and a control group in patients with acute coronary syndrome who were already being treated with statins. The random-effect model was used to measure the pooled effect, which was presented in terms of mean difference, standardized mean difference, and risk ratio.

**Results:**

Acquired 12 studies that fulfilled our criteria. The addition of PCSK9 inhibitors ameliorates the plaque phenotype significantly in terms of percent atheroma volume (*P* = 0.02), total atheroma volume (*P* < 0.010), fibrous cap thickness (*P* < 0.00001), lipid arc (*P* < 0.00001), quantitative flow ratio (*P* = 0.003), and diameter of stenosis (*P* = 0.0003) but not in lipid/lesion length (*P* = 0.17). The administration of PCSK9 inhibitors led to a considerable improvement in all lipid profiles (*P* < 0.00001). Regarding safety analysis, there is no substantial disparity in the likelihood of non-serious side events (RR 1.21; *P* = 0.2), however, a significant reduction in the risk of serious adverse effects (RR 0.77; *P* = 0.04) in the PCSK9 inhibitor group.

**Conclusions:**

The addition of PCSK9 inhibitors compared to statin-only treatment led to a majority of patients experiencing significant benefits in terms of safety and efficacy following ACS.

**Supplementary Information:**

The online version contains supplementary material available at 10.1186/s43044-024-00567-2.

## Background

Acute Coronary Syndrome (ACS) is a notable cardiovascular issue due to its substantial impact on morbidity and mortality rates [1]. Ischemic heart disease is responsible for almost 50% of deaths caused by cardiovascular diseases. Moreover, ischemic heart disease is responsible for around 12% of the total number of disability-adjusted life-years (DALYs) lost globally annually [2]. Fortunately, the progress in contemporary medicine has resulted in enhanced results for individuals suffering from coronary heart disease. An example of enhancement is the optimization of the administration and prevention of acute coronary syndrome [3]. According to postmortem studies conducted in the 1980s, the prevailing belief is that the main cause of fatal myocardial infarction in patients with pre-existing coronary artery disease (CAD) is the rupture of atherosclerotic plaques [4]. Therefore, maximizing the utilization of lipid-lowering medications may be a viable approach to tackle the difficulties faced by those who already have or are prone to acute coronary syndrome. Prior studies have shown that there is a direct correlation between the overall exposure to LDL-C and the incidence of clinical events. According to a recent meta-analysis conducted by Koskinas et al., it was estimated that a decrease of 1 mmol/l (38.67 mg/dL) in LDL-C is associated with a reduction of roughly 19% in the risk of cardiovascular events [3, 5].

Statins are currently recognized as the primary treatment for reducing LDL-C levels and stabilizing plaque in individuals with acute coronary syndrome (ACS), provided there are no contraindications [1, 5]. However, a significant proportion of patients are unable to achieve adequate reductions in LDL-C levels or tolerate the prescribed doses of statins. As a result, it is necessary to investigate alternative therapies. Proprotein convertase subtilisin/kexin type 9 (PCSK9) inhibitors have emerged as significant pharmacological agents for reducing cholesterol levels, often used in conjunction with statins and/or ezetimibe. They function by reducing the degradation of LDL receptors and improving the removal of LDL-C [3]. Currently, two PCSK9 monoclonal antibodies (PCSK9-mAbs), namely alirocumab and evolocumab, are commonly utilized in clinical practice. Prior meta-analyses conducted by Guedeney et al. in 2021 and 2022 have demonstrated that alirocumab and evolocumab are advantageous in lowering major cardiac adverse events (MACE), including acute coronary syndrome (ACS), stroke, and coronary revascularization. These studies specifically targeted patients who had dyslipidemia and/or a pre-existing condition of atherosclerotic cardiovascular disease (ASCVD). The investigation also showed that the use of PCSK9 inhibition with these medications had a favorable safety profile [6, 7]. However, based on our present understanding, there is still an absence of studies assessing the safety and effectiveness of PCSK9 inhibitors and examining the direct impact of statin and PCSK9 inhibitors on plaque phenotype in specific groups of patients who established ACS.

Hence, the objective of this meta-analysis was to comprehensively investigate the effect of PCSK9 inhibitor combined with statins compared to statin-only treatment in terms of altering lipid profile as well as evaluating the effect of lipid profile changes by evaluating the coronary plaque phenotype on patients following acute coronary syndrome. We also evaluate the safety of the PCSK9 addition in terms of adverse events evaluated.

## Method

This systematic review and meta-analysis were conducted using the standards provided by the Preferred Reporting Items for Systematic Reviews and Meta-analysis (PRISMA), which may be found at http://www.prisma-statement.org/. See the supplementary material, specifically Table S1, for the fully completed PRISMA 2020 checklist of this investigation. The study protocol has been registered on the International Prospective Register of Systematic Reviews (PROSPERO) with the registration number CRD42023494415. The registration can be seen at https://www.crd.york.ac.uk/prospero/.

### Literature search

Four writers (DIH, MY, NA, FRQ) conducted individual searches of papers from PubMed, Cochrane Central Register of Controlled Trials (CENTRAL), and ProQuest using Boolean operators, namely: ("proprotein convertase subtilisin kexin type 9 inhibitor" OR "PCSK9 inhibitors" OR "alirocumab" OR "evolocumab") AND "Statin" AND ("acute coronary syndrome" OR "myocardial infarction") AND (("Plaque" OR "Atheroma" OR "fibroatheroma") OR ("Safety" OR "Efficacy")). The authors incorporated several Medical Subject Headings (MeSH) and supplementary free-text phrases to generate search terms that are pertinent to the database. The determination of inclusion and exclusion criteria was conducted prior to the literature search, following the PICO methodology (Table 1). There are no limitations or constraints on the utilization of language during the process of doing the data search. Furthermore, the writers have assembled an extensive collection of references derived from the included studies. Afterwards, the acquired results are eliminated of any duplicates and validated based on the eligibility requirements.

**Table 1:** PICO framework

PICO	Keywords
Patient	“Patients following ACS / MI”
Intervention	“Proprotein convertase subtilisin kexin type 9 inhibitor” OR “PCSK9 Inhibitors” OR “Alirocumab” OR “Evolocumab”
Comparison	“Statin”
Outcome	“Plaque” OR “Atheroma” OR “Fibroatheroma”
	“Safety” OR “Efficacy”

*ACS*: Acute coronary syndrome; *MI*: Myocardial Infarction; *PCSK9*: Proprotein convertase subtilisin kexin type 9 inhibitor

### Study eligibility criteria

The identification of publications was independently examined and selected by four reviewers, with the first reviewer completing the final examination to determine eligibility. Furthermore, the studies underwent a rigorous screening method based on predetermined criteria for determining which ones would be included and excluded. Regarding inclusion criteria, (a) the trials encompassed adult patients aged 18 and above who had experienced ACS. (b) Comparative studies examining the effects of PCSK9 inhibitors against non-PCSK9 inhibitors in patients who are already receiving statin treatment. (c) Research studies that assess the effectiveness, safety, and characteristics of plaque in relation to the final outcome. (d) Recent full-text studies published within the past 10 years that contain readily available data. The exclusion criteria were (a) Not accessible papers. (b) Non-human clinical trial. (c) Studies with unreported predetermined outcomes.

### Study selection

Four investigators (DIH, FRQ, MY, NA) independently evaluated all literature search results, including screening titles and abstracts based on predefined eligibility criteria. The study's search results from numerous databases were saved in a Google Sheets document located at the following URL: https://docs.google.com/spreadsheets/ (Google, Mountain View, CA, USA). Each investigator carried out an individual and comprehensive assessment of the available literature. When confronted with studies that are unclear or puzzling, the next course of action is to participate in a transparent discussion among researchers. Following that, a thorough analysis of the entire text was carried out to exclude publications that did not fit the pre-established criteria for inclusion. All studies were validated and verified by all investigators. Afterwards, a data extraction table was created to consolidate the data obtained from the selected studies.

### Data extraction

Three investigators (DIH, MY, NA) conducted individual assessments of all study titles and abstracts. Any disagreements among the investigators will be addressed at a later stage, after all duplicated studies have been resolved. The data were acquired by employing a specified reporting form. The chosen studies were transferred to a Google sheet and thereafter evaluated for their suitability through a process of deliberation and consultation with the senior author (IP, AMA) until a consensus was reached. When choosing a publication from various sources that discuss the same studies, we gave priority to the one with the highest number of participants and the most recent publication date. The following information was extracted from eligible studies: author's name, year of publication, trial protocol (if any), study design, number of samples, sample characteristics, treatment and the doses given, follow-up duration, and endpoints. If there was any missing data, the corresponding authors were contacted.

### Quality assessment

The assessment of bias was conducted by DIH and FRQ, under the supervision of the other authors, using the Modified Jadad Scale score for trials and the Newcastle–Ottawa Scale (NOS) for observational studies. The Modified Jadad Scale, ranging from 0 to 8, was used to evaluate the risk of bias in randomized studies [8]. A good quality study is defined by a score of 4 or greater [9]. Regarding NOS, studies of high quality were determined by receiving a rating of 3 or 4 stars in the selection domain, 1 or 2 stars in the comparability domain, and 2 or 3 stars in the outcome/exposure domain.

### Quantitative analysis

A quantitative pooled meta-analysis is conducted when there is data from multiple studies with similar measurements and outcomes. The meta-analysis was conducted using RevMan 5.4 (The Cochrane Collaboration, The Nordic Cochrane Centre, Copenhagen, Denmark). Inverse Variance method was used to obtain mean difference (MD) and its standard deviation (SD). The Q test was employed to assess heterogeneity, and the results were shown with *I*^2^ values. *I*^2^ values ranging from 0 to 40% indicate a low level of heterogeneity. Values between 30 and 60% are considered to represent moderate heterogeneity. When the *I*^2^ value falls between 50 and 90%, it indicates substantial heterogeneity, meaning that at least half of the variation in effect sizes is due to genuine differences between studies. Finally, *I*^2^ values of 75–100% indicate considerable heterogeneity [10]. The pooled effects were assessed using a random-effects model to account for the variability in study designs and the large range of potential treatment effect sizes across studies. However, a fixed-effects model was employed where there was no substantial heterogeneity between studies [11]. Mean difference (MD) or standardized mean difference (SMD) and the corresponding 95% confidence interval (CI) of the continuous endpoints were calculated to compare the efficacy of both treatments. The safety endpoints were quantitatively analyzed as risk ratio (RR) and the 95% CI. A p-value of < 0.05 was considered statistically significant. Sensitivity and subgroup analyses were conducted to explore the heterogeneity and robustness of the result where possible, especially between alirocumab and evolocumab. To find the publication bias, the authors utilized a mix of Egger’s and Begg’s tests. Egger’s and Begg’s tests revealed publication bias if the *P* value of < 0.05 [12]. Test for funnel plot asymmetry is also used when there are at least 10 eligible studies included [10]. Additionally, we conducted a sensitivity analysis of our meta-analysis using the leave-one-out approach to determine the source of heterogeneity as well as to assess the reliability of our results. A leave-one-out forest plot was computed using Stata/MP 18 for Mac (StataCorp, TX, USA).

### Type of outcome measures

There were two primary outcomes comprised of efficacy represented by plaque phenotype and lipid profile between the two groups. The first primary endpoints include efficacy outcomes represented by plaque phenotype including percent atheroma volume (PAV), total atheroma volume (TAV), fibrous cap thickness (FCT), Mean Total Lipid Core Burden Index (LCBI), Lipid Arc (LA), Quantitative Flow Ration (QFR), Diameter of Stenosis (DoS), and lipid/lesion length changes evaluated either using intravenous ultrasonography (IVUS) or optical coherence tomography (OCT) or angiography evaluating coronary physiological function (Quantitative Coronary Angiography (QCA)). Another primary endpoint are the changes in lipid profile including LDL cholesterol, total cholesterol, and triglycerides value. The secondary endpoint comprises treatment-related adverse effects divided by non-serious adverse effects (e.g., injection reaction, myalgia, etc.) and serious adverse effects (e.g., Re-myocardial infarction, ischemic stroke, death) (non-specified).

## Results

### Study selection and identification

**Fig. 1:**
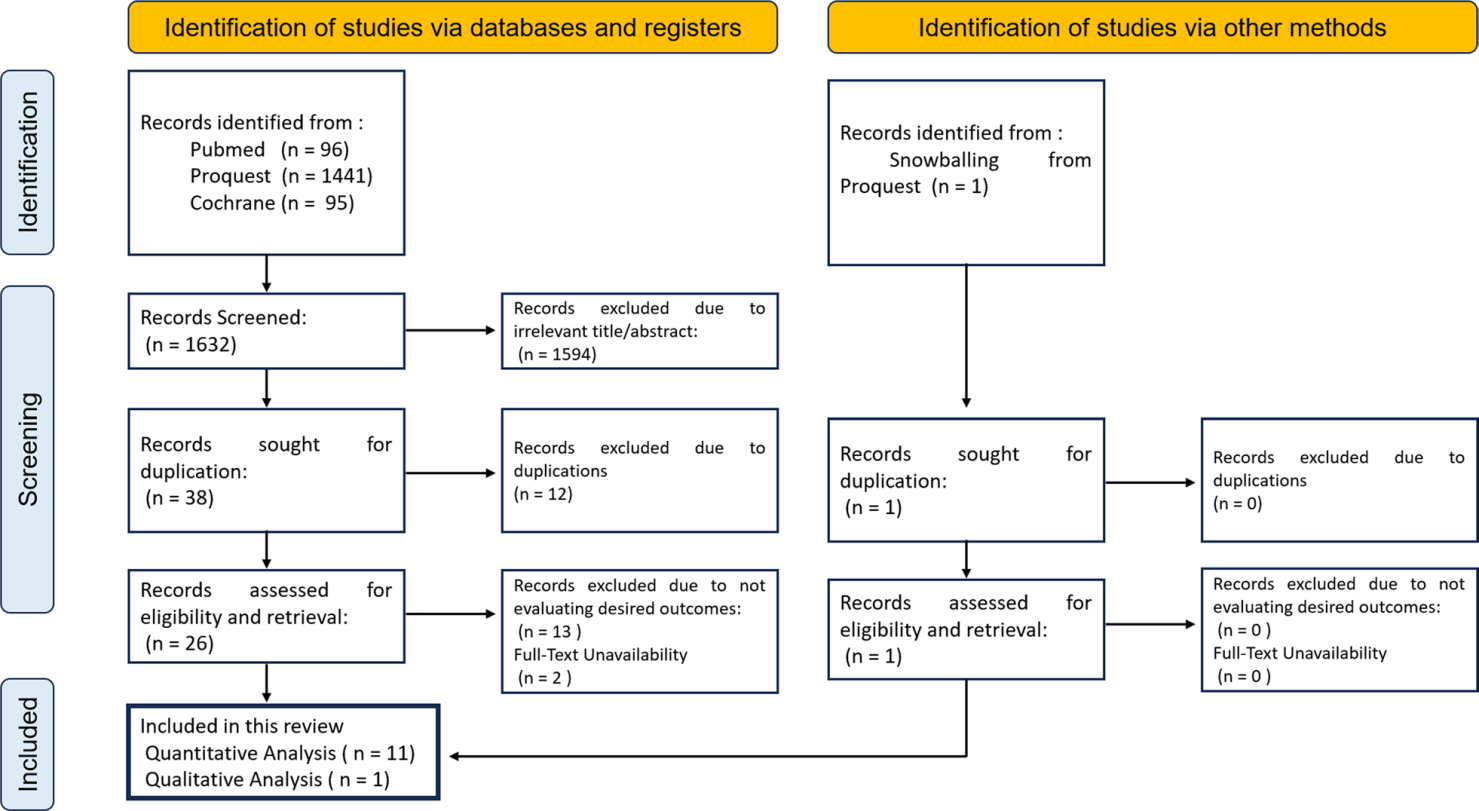
Diagram of study selection using PRISMA flowchart

The PRISMA study flowchart is shown in Fig. 1. By employing a literature search strategy, the initial search of the accessible databases yielded 1,632 titles and abstracts that were published over the past decade. These results were obtained from three databases and one additional source, which was identified by snowballing. Subsequently, a total of 1,594 articles were eliminated from consideration due to titles or abstracts that were not relevant. After conducting a thorough examination, the authors identified 38 studies that would undergo further screening. During the screening procedure, the authors identified 12 duplicated research, 13 studies that did not yield the expected outcomes, and 2 papers that were not accessible for a full-text review. In this process, the authors include 11 studies as well as one study identified through snowballing (a total of 12 studies) due to insufficient data and/or inaccessibility of the other research. In this analysis, the authors incorporate a total of nine randomized controlled trial studies and three cohort studies. Six research assessed the phenotypic characteristics of plaques, whereas nine studies evaluated lipid profiles. Subsequently, seven studies examined the occurrence of adverse events. This study covered all recruited cases, which consisted of 20,964 individuals from 12 studies.

### Risk of bias assessment

**Fig. 2:**
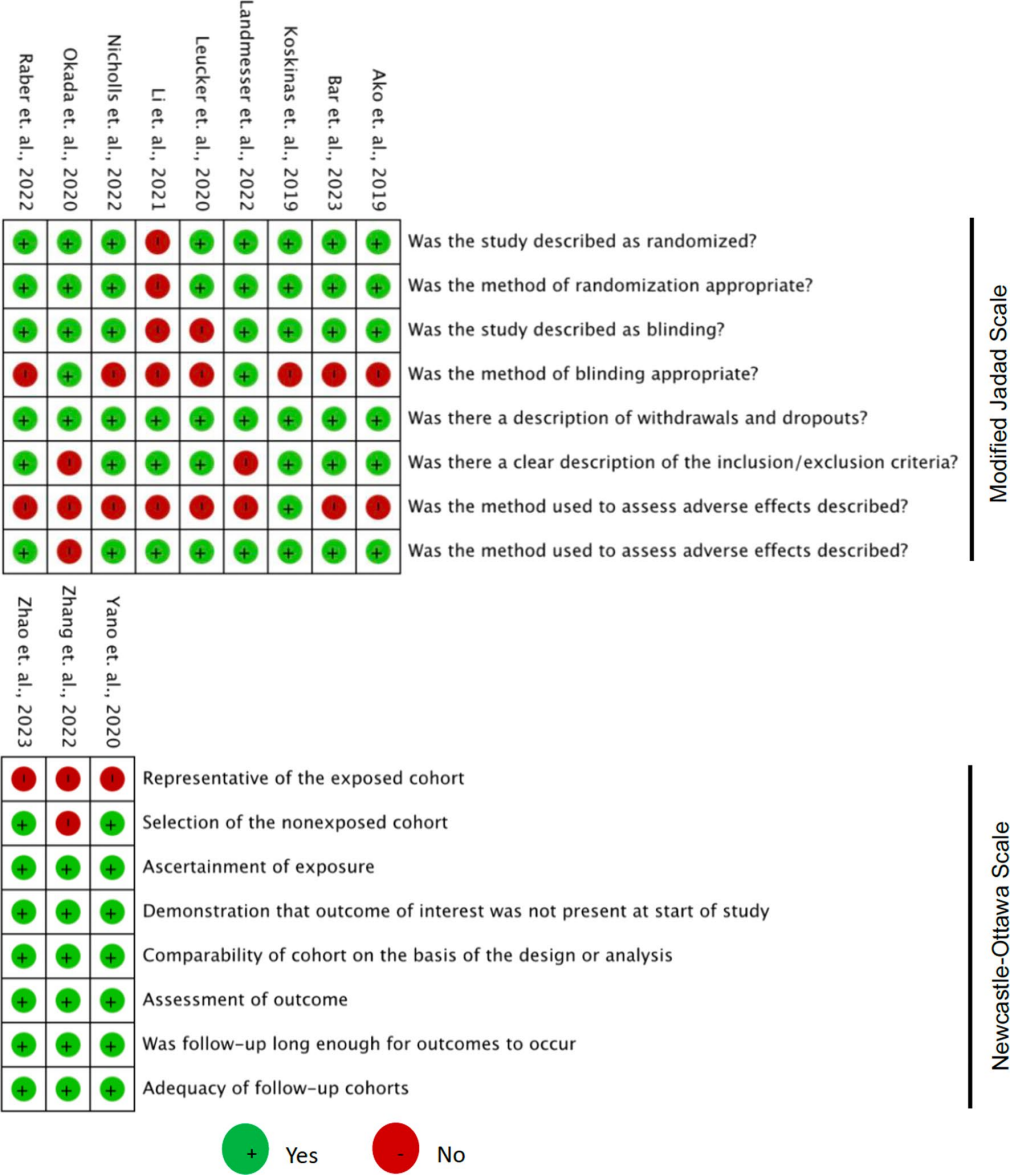
Quality assessment of the risk of bias assessment of 9 controlled trials and 3 observational studies (cohort)

The last column of Table 2 shows the results of the quality assessment of the included trial and observational studies, while Fig. 2 shows the detail points of the Modified Jadad Scale and Newcastle–Ottawa Scale for each study that was included in this systematic review and meta-analysis. Most of the trial studies have good quality except for the study of Li *et. al.,* and most of the observational studies have good quality except study from Zhang *et. al.* with fair quality [9].

**Table 2:** Characteristics of studies

Author	Year	Study design	PCSK9-inhibitors	Control	Follow up (weeks)	Study quality
Raber et al	2022	RCT, Double-Blind, Multicenter	Alirocumab 150 mg q2w + Rosuvastatin 20 mg qd	Rosuvastatin 20 mg qd + Placebo	52	High
Nicholls et al	2022	RCT, Double-Blind, Multicenter	Evolocumab 420 mg qm + Statin (Unspecified)	Statin (Unspecified) + Placebo	50	High
Koskinas et al	2019	RCT, Double Blind, Multicenter	Evolocumab 420 mg qm + Atorvastatin 40–80 mg mg qd	Atorvastatin 40–80 mg qd + Placebo	8	High
Ako et al	2019	RCT, Double-Blind, Multicenter	Alirocumab 75 mg q2w + Atorvastatin = > 10 mg/day or Rosuvastatin = > 5 mg/day	Atorvastatin = > 10 mg/day or Rosuvastatin = > 5 mg/day + Placebo	36	High
Bar et al	2023	RCT, Double-Blind, Multicenter	Alirocumab 150 mg q2w + Rosuvastatin 20 mg qd	Rosuvastatin 20 mg qd + Placebo	52	High
Li et al	2021	Non-randomized Controlled Trial Single Center	Alirocumab 140 mg q2w + Rosuvastatin 10 mg qn	Rosuvastatin 10 mg qn	8	Low
Okada et al	2020	RCT, Single Center	Evolocumab 140 mg q2w + Pitavastatin 2 mg qd	Pitavastatin 2 mg qd	4	High
Landmesser et al	2022	RCT, Double Blind, Multicenter	Alirocumab 75 mg q2w + Atorvastatin 40–80 mg qd or Rosuvastatin 20–40 mg qd	Atorvastatin 40–80 mg qd or Rosuvastatin 20–40 mg qd + Placebo	146	High
Leucker et al	2020	RCT, Placebo-Controlled, Single-Center	Evolocumab 420 mg qm + High-intensity statin (Unspecified)	High-intensity statin (Unspecified) + placebo	4	High
Zhao et al	2023	Cohort Prospective	Evolocumab 140 mg q2w + Rosuvastatin 20 mg qd	Rosuvastatin 20 mg qd + Placebo	52	Good
Zhang et al	2022	Single-center, Cohort Retrospective	Evolocumab 140 mg q2w + atorvastatin 20 mg—40 mg qd or rosuvastatin 10 mg—20 mg) qd or combination with ezetimibe	atorvastatin 20 mg—40 mg qd or rosuvastatin 10 mg—20 mg) qd or combination with ezetimibe	18	Fair
Yano et al	2020	Cohort Retrospective	Evolocumab 140 mg qw2 + Rosuvastatin 5 mg qd	Rosuvastatin 5 mg qd	12	Good

*RCT*: Randomized controlled trial; *mg*: milligram; *q2w*: every two weeks: *qd*: once daily; *qm*: once in a month/monthly

### Summary of included studies

Included in the analysis were nine randomized controlled trials and three observational studies, including a total of 20,964 participants. The authors conducted a quantitative analysis of 11 out of the 12 investigations. The studies encompassed patients with acute coronary syndrome (ACS), including unstable angina, non-ST-segment elevation myocardial infarction (NSTEMI), or ST-segment elevation myocardial infarction (STEMI). The treatment arm consisted of either alirocumab or evolocumab in combination with a high-intensity statin. In all investigations, the control arm received a high-intensity statin. The dosage of alirocumab varied between 75 and 150 mg, administered every 2 weeks to once a month. In contrast, the dosage of evolocumab ranged from 140 to 420 mg, given every 2 weeks to once a month. The duration of follow-up varies across research, ranging from around 4 to 146 weeks. The specifics of the attributes of the studies that were included are outlined in Table 2.

### Primary outcome: plaque phenotype

**Fig. 3:**
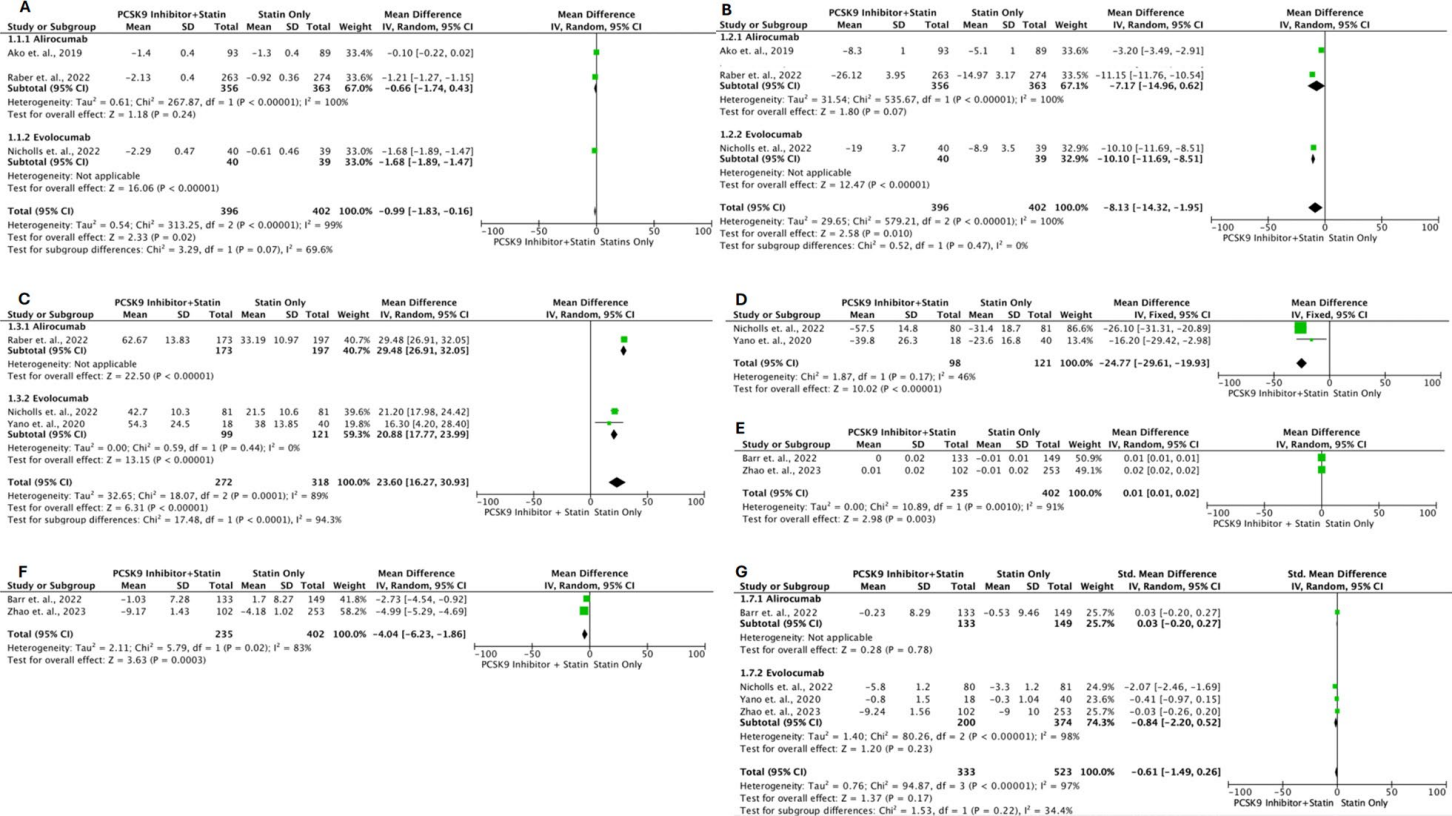
Forest plots illustrate the effects of PCSK9 additions in statin-treated ACS patients on plaque phenotype between baseline and follow-up. (**A:** Percent Atheroma Volume (PAV); **B:** Total Atheroma Volume (TAV); **C:** Fibrous Cap Thickness (FCT); **D:** Lipid Arc (LA); **E:** Quantitative Flow Ration (QFR); **F:** Diameter of Stenosis (DoS); **G:** Lipid/Lesion Length)

Several outcomes were measured related to *plaque phenotype*. Figure 3A describes the outcome of the PAV change evaluated with IVUS. Three studies (Ako et al*.,* 2019; Nicholls et al*.,* 2022; and Raber et al*.,* 2022) [13–15] with a total of 798 patients (396 in the intervention group and 402 in the control group), found that the addition of PCSK9 inhibitors to statin medication resulted in a more substantial decrease in PAV compared to statin therapy alone (MD: − 0.99, 95% CI − 1.83, − 0.16; *P* = 0.02; *I*^2^ = 99%). Based on the analysis of subgroup differences between alirocumab and evolocumab, there is no statistically significant distinction (P = 0.07). The findings of the TAV modification measured by IVUS are depicted in Fig. 3B. Three studies (Ako et al*.,* 2019; Nicholls et al*.,* 2022; and Raber et al*.,* 2022) [13–15] with a total of 798 patients (396 in the intervention group and 402 in the control group), have found that the addition of PCSK9 inhibitors to statin medication results in a more significant decrease in TAV compared to statin therapy alone (MD: -8.13; 95% CI − 14.32, -1.95; P < 0.010; *I*^2^ = 100%). Based on the analysis of subgroup differences between alirocumab and evolocumab, no statistically significant difference was found (P = 0.47).

On FCT changes evaluated by OCT, the results are described in Fig. 3C. Three studies (Nicholls et al*.,* 2022; Raber et al*.,* 2022; and Yano et al*.,* 2020) [14–16] with the total patients of 590 (272 intervention vs 318 control) reported that addition of PCSK9 inhibitors to statin therapy increases FCT more significant than statin-therapy alone (MD: 23.60; 95% CI 16.27, 30.93; *P* < 0.00001; *I*^2^ = 89%). From subgroup differences between alirocumab and evolocumab, there is significant difference (*P* < 0.0001). On LA changes evaluated by OCT, the results are described in Fig. 3D. Two studies (Nicholls et al*.,* 2022 and Yano et al*.,* 2020) [14, 16] with the total patients of 219 (98 intervention vs 121 control) reported that addition of PCSK9 inhibitors to statin therapy decreases LA more Significant than statin-therapy alone (MD: − 24.77; 95% CI − 29.61, − 19.93; *P* < 0.00001; *I*^2^ = 46%).

On QFR changes evaluated by software based on 3D angiography data, the results are described in Fig. 3E. Two studies (Barr et al*.,* 2023 and Zhao et al*.,* 2023) [17, 18] with the total patients of 637 (235 intervention vs 402 control) reported that addition of PCSK9 inhibitors to statin therapy increase QFR more significant than statin-therapy alone (MD: 0.01; 95% CI 0.01, 0.02; *P* = 0.003; *I*^2^ = 91%). On DoS changes evaluated by coronary physiological function based on 3D angiography, the results are described in Fig. 3F. Two studies (Barr et al*.,* 2023 and Zhao et al*.,* 2023) [17, 18] with the total patients of 637 (235 intervention vs 402 control) reported that addition of PCSK9 inhibitors to statin therapy decrease percent diameter of stenosis more significant than statin-therapy alone (MD: − 4.04; 95% CI − 6.23, − 1.86; *P* = 0.0003; *I*^2^ = 83%).

On Lipid/lesion length changes evaluated by coronary physiological function based on 3D angiography and OCT, the results are described in Fig. 3G. Four studies (Barr et al*.,* 2023; Nicholls et al*.,* 2022; Zhao et al*.,* 2023; and Yano et al*.,* 2020**)** [14, 16–18] with the total patients of 856 (333 intervention vs 523 control) reported that addition of PCSK9 inhibitors to statin therapy decrease lipid/lesion length more but not significant than statin-therapy alone (SMD: − 0.61; 95% CI − 1.49, 0.26; *P* = 0.17; *I*^2^ = 97%). From subgroup differences between alirocumab and evolocumab, there is no significant difference (*P* = 0.22).

### Primary outcome: lipid profile

**Fig. 4:**
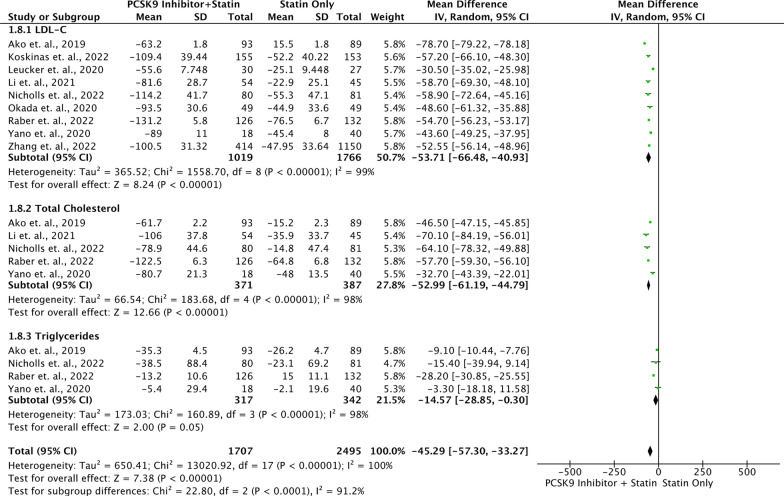
Forest plot of lipid profile amelioration in comparing between population with PCSK9 inhibitor and statin vs. population with statin medication only

The administration of both statins and PCSK9 inhibitors in combination therapy resulted in a significant reduction (*P* < 0.05) in various lipid markers, including total cholesterol (TC), LDL-C, apolipoprotein B (Apo B), triglycerides (TG), non-high-density lipoprotein cholesterol (non-HDL-C), and lipoprotein a. Additionally, this combination therapy led to a significant increase in levels of HDL-C and Apo-A1 (Table 3). The forest plot in Fig. 4 included routine lipid test parameters such as LDL-C, TC, and TG from nine studies involving 1,707 patients who were taking PCSK9 inhibitors in addition to statin therapy, and 2495 patients who were only taking statin medication. The results showed that the group of patients who took PCSK9 inhibitors along with statin had a more favorable improvement in their lipid profile values, with a decrease in LDL-C, TC, and TG levels (MD: − 45.29; 95% CI − 57.30, − 33.27; *P* < 0.00001; *I*^2^: 100%). The qualitative analysis conducted by Landmesser et al. [19] based on a sub-analysis of the ODYSSEY study, provides further support for previous quantitative findings. The study involved 17,589 patients with a history of acute coronary syndrome (ACS) and hyperlipidemia who were not taking ezetimibe. The results showed that treatment with alirocumab, a statin therapy, enabled 94.6% of patients to reach the treatment goal outlined in the 2019 European guideline. The LDL-C levels of less than 1.4 mmol/L (less than 54.14 mg/dL) were seen in 85.2% of the patients who experienced recurrent cardiovascular events. Furthermore, a majority of these patients, specifically 85.2%, achieved LDL-C levels below 1.0 mmol/L (below 38.67 mg/dL) [19].

**Table 3:** Results of lipids profile analysis

Lipid Profile	Included Studies	*I*^2^	MD	95% CI	*P* value
Total Cholesterol (mf/dL)	5	Random	− 52.99	− 61.19 – (− 44.78)	< 0.00001*
LDL-C (mg/dL)	9	Random	− 53.71	− 66.48 – (− 40.94)	< 0.00001*
HDL-C (mg/dL)	5	Random	3.34	3.15 – 3.54	< 0.00001*
Non-HDL-C (mg/dL)	3	Random	− 50.4	− 50.94 – (− 49.85)	< 0.00001*
TG (mg/dL)	4	Random	− 14.57	− 28.85 – (− 0.30)	0.05*
Apolipoprotein AI (mg/dL	3	Random	7.28	5.78 – 8.78	< 0.00001*
Lipoprotein (a) (mg/dL)	3	Random	− 5.1	− 9.39 – (− 80)	0.02*
Apolipoprotein B (mg/dL)	4	Random	− 14.36	− 20.02 – (− 8.70)	< 0.00001*

*LDL-C*: Low density lipoprotein cholesterol; *TC*: Total cholesterol; *HDL-C*: High density lipoprotein cholesterol; *TG*: Triglycerides; *Apo-A1*: Apolipoprotein A1; *Non-HDL-C*: Non-high-density lipoprotein cholesterol; *Lp(a)*: Lipoprotein a; *ApoB*: Apolipoprotein B

### Secondary Outcome: Adverse effects

**Fig. 5:**
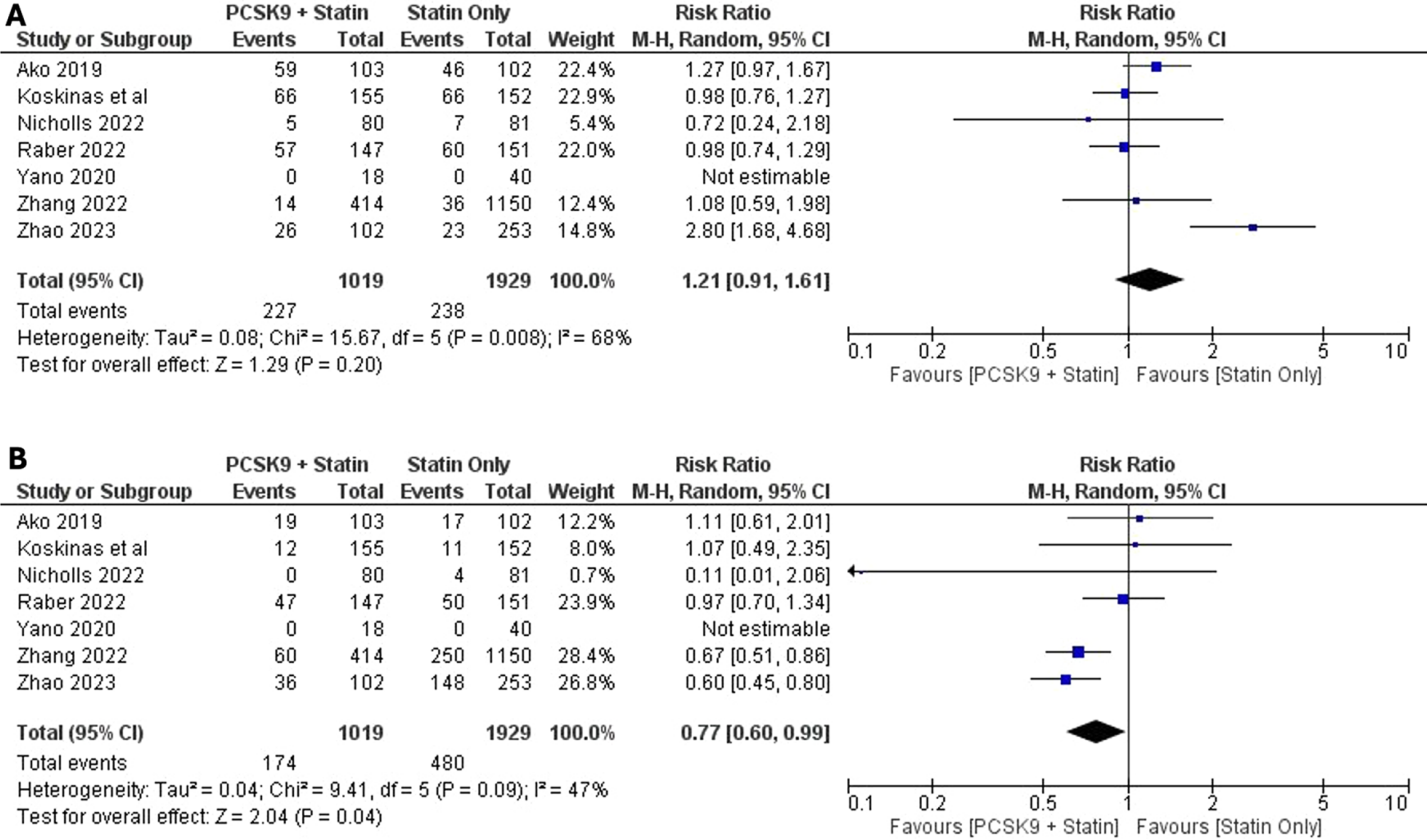
Forest plots illustrate the effects of PCSK9 additions in statin-treated ACS patients on adverse effects. (**A:** Non-Serious Adverse Effects; **B:** Serious Adverse Effects)

Adverse effects were categorized as non-serious adverse effects and serious adverse effects. Several studies (Ako et al*.,* 2019; Barr et al*.,* 2023; Koskinas et al*.,* 2019; Landmesser et al*.,* 2022; Li et al*.,* 2021; Nicholls et al*.,* 2022; Okada et al*.,* 2020; Raber et al*.,* 2022; Zhao et al*.,* 2023**)** [5, 13–15, 17–21] assessed both serious and mild adverse effects with varying outcomes, and the findings from these evaluations are depicted in Fig. 5. Among the 2,948 participants (1,019 in the intervention group and 1,929 in the control group), there is no statistically significant disparity in non-serious side effects between the two groups (Risk Ratio: 1.21; 95% CI 0.91, 1.61; *P* = 0.2; *I*^2^ 68%). Nonetheless, in serious adverse effects comparisons were found a significant difference between groups (Risk ratio: 0,77; 95% CI 0.60–0.99; *P* = 0.04; *I*^2^ 47%). The summary of the endpoints is summarized in Table 4.

**Table 4:** Summary of endpoints

Plaque phenotype changes									
Endpoints	Number of studies	Model	MD	95% CI	*P*	pHet	*I*^2^ (%)	P Begg	P Egger
**PAV**	3	Random	− 0.99	− 1.83 to − 0.16	0.02*	< 0.00001	99%	1	0.88
**TAV**	3	Random	− 8.13	− 14.32 to -1.95	0.010*	< 0.00001	100%	1	0.6
**FCT**	3	Random	23.6	16.27 to 30.93	< 0.00001*	0.0001	89%	1	0.69
**LA**	2	Random	− 24.77	− 29.61 to − 19.93	< 0.00001*	0.17	46%	N/A	N/A
**QFR**	2	Random	0.01	0.01 to 0.02	0.003*	0.0010	91%	N/A	N/A
**DoS**	2	Random	− 4.04	− 6.23 to − 1.86	0.0005*	0.05	74%	N/A	N/A
**Lipid/lesion length**	4	Random	− 0.61	− 1.49 to 0.26	0.17	< 0.00001	97%	1	0.13

Lipid profile changes									
Endpoints	Number of studies	Model	MD	95% CI	*P*	pHet	*I*^2^ (%)	P Begg	P Egger
**Total cholesterol (mg/dL)**	5	Random	− 52.99	− 61.19 to − 44.78	< 0.00001*	< 0.00001	98%	1	0.46
**LDL-C (mg/dL)**	9	Random	− 53.71	− 66.49 to − 40.94	< 0.00001*	< 0.00001	99%	0.17	0.03
**HDL-C (mg/dL)**	5	Random	3.34	3.15 to 3.54	< 0.00001*	0.69	0%	0.46	0.06
**non-HDL-C (mg/dL)**	3	Random	− 50.4	− 50.94 to − 49.85	< 0.00001*	< 0.00001	99%	1	0.55
**TG (mg/dL)**	4	Random	− 14.57	− 28.85 to − 0.3	0.04*	< 0.00001	98%	0.73	0.71
**Apolipoprotein AI (mg/dL)**	3	Random	7.28	5.78 to 8.78	< 0.00001*	0.0007	86%	1	0.79
**Lipoprotein (a) (mg/dL)**	3	Random	− 5.1	-9.39 to -0.80	0.02*	< 0.00001	100%	0.29	0.24
**Apolipoprotein B (mg/dL)**	4	Random	− 14.36	-20.02 to -8.70	< 0.00001*	< 0.00001	100%	0.09	0.01

Adverse effects									
Endpoints	Number of studies	Model	Risk Ratio	95% CI	*P*	pHet	*I*^2^ (%)	P Begg	P Egger
**Non-Serious Adverse Effects**	7	Random	1.21	0.91 to 1.61	0.2	0.008	68%	0.7	0.61
**Serious Adverse Effects**	7	Random	0.77	0.60 to 0.99	0.04*	0.09	47%	1	0.7

*PAV*, percent atheroma volume. *TAV*, total atheroma volume. *FCT*, fibrous cap tissue. *LA*, lipid arc. *QFR*, quantitative flow ratio. *DoS*, diameter of stenosis. *LDL-C*, low-density lipoprotein cholesterol. *HDL-C*, high-density lipoprotein cholesterol. *TG*, triglyceride. *MD*, mean difference. *SMD*, standardized mean difference. *CI*, confidence interval

*Significant

### Heterogeneity and publication bias

Significant heterogeneity of effect was observed in the majority of endpoints. All endpoints regarding plaque phenotype except for LA endpoint have significant heterogeneity, as well as lipid profile changes endpoints except for HDL-C endpoint. Both adverse effect endpoints have significant heterogeneity. Examination of the inverted funnel plot demonstrated symmetry, suggesting that there was no significant publication of bias, as the p-values for the Begg’s and Egger’s tests were ≥ 0.05 for all endpoints except for the LDL-C change endpoint.

### Sensitivity analysis

**Fig. 6:**
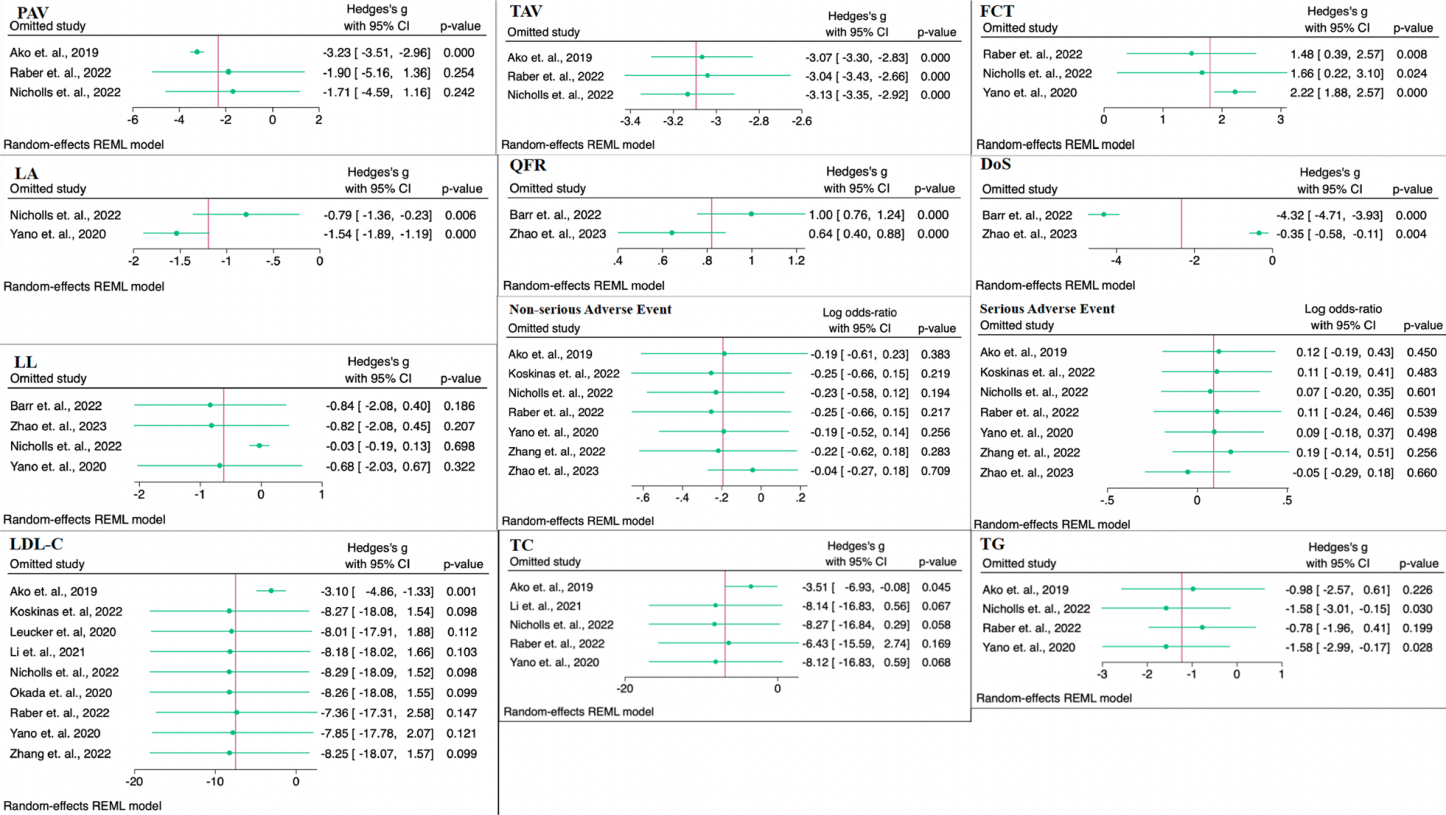
Forest plot of leave-one-out method for sensitivity analysis

The leave-one-out approach was conducted in this meta-analyses by systematically omitting one study at a time during each analysis to assess the influence of each individual study on the pooled effect size. In this study, the leave-one-out sensitivity analysis were conducted in all endpoints/outcomes. The results demonstrated that the pooled correlation coefficient remained robust and unaffected by any individual study in several outcomes/endpoints namely TAV, FCT, Lipid Arc (LA), QFR, and DoS. At the same time, their heterogeneity in meta-analysis showed a moderate to high. As shown in Fig. 6, the statistical significance of the results remains unchanged when any individual research utilized in any endpoints meta-analysis [TAV, FCT, Lipid Arc (LA), QFR, and DoS] is excluded. The result of the sensitivity test performed using the leave-one-out approach confirm the robustness of the study conclusions. However, certain endpoints/outcomes including PAV, lipid profile, and adverse events showed that certain studies outperformed others included in the study. Therefore, excluding these studies from the analysis significantly alters the overall number being an insignificant result.

## Discussion

This systematic review and meta-analysis study aims to assess the effectiveness of PCSK9 inhibitors as plaque stabilizers for secondary prevention following acute coronary syndrome (ACS). ACS refers to a range of diseases that involve patients who have recently had changes in their clinical symptoms or indications. These changes may or may not be accompanied by changes in their 12-lead electrocardiogram (ECG) and may or may not be accompanied by acute increases in cardiac troponin (cTn) levels [22]. Despite significant advancements in diagnosing and treating acute coronary syndromes, cardiovascular disease continues to be the primary cause of death globally, with approximately half of these fatalities attributed to ischemic heart disease [23]. Implementing secondary prevention measures following an acute coronary syndrome (ACS) is crucial for enhancing the overall well-being of patients and reducing the occurrence of morbidity and mortality. It is advisable to commence this as soon as feasible following the initial occurrence [22]. Secondary prevention includes a range of non-pharmacological measures, including advice on nutrition and exercise, smoking cessation, and cardiac rehabilitation, in addition to pharmacological therapies [23]. Regarding recent advancements in secondary prevention medical therapy, there is significant new research that provides guidance on the use of lipid-lowering and anti-inflammatory medications. Lowering the levels of atherogenic lipoproteins in the bloodstream significantly reduces the likelihood of experiencing adverse cardiovascular events in many clinical cases [23]. A currently published meta-analysis by Ahmed Atia, *et. al.* demonstrated that the administration of PCSK9 inhibitors, namely alirocumab and evolocumab, for secondary prevention in 24,732 patients following ACS resulted in notable improvements in lipid profiles. These improvements included significant reductions in LDL-C, TC, TG, Lipoprotein A, and Apolipoprotein B levels. Furthermore, the conducted study revealed a notable elevation in HDL-C and Apolipoprotein-A1 concentrations [24]. The investigation revealed that enhancements in the lipid profile led to a decrease in the occurrence of myocardial infarction and cerebrovascular events. Therefore, the study in this meta-analysis is in accordance with strengthen the results of previous stud by analyzing the efficacy of PCSK9 inhibitors combined with statins in patients following ACS by further reviewing the amelioration effect on lipid profile and also on the terms of direct effect on the plaque/atheroma phenotype.

A meta-analysis has shown that the reduction in atherosclerotic cardiovascular disease (ASCVD) is directly related to the dosage of LDL-C-lowering medications [5]. As the absolute reduction in LDL-C increases, the cardiovascular risk decreases proportionally. The advantages linked to the reduction of LDL-C are not limited solely to statin medicine. There is no defined level of LDL-C at which the beneficial benefits cease or the harmful consequences start. Previous research mostly examined the effects of statins and ezetimibe on reducing LDL-cholesterol (LDL-C) levels after acute coronary syndromes. However, there is now emerging evidence that additional lipid-lowering drugs, like PCSK9 inhibitors, are showing promising results [23]. The current guidelines establish a target LDL-C level of < 55 mg/dL for secondary prevention following an acute coronary syndrome (ACS) occurrence [22]. While monotherapy successfully achieves LDL-C goals in a majority of individuals, a considerable number of high-risk patients or those with extremely high LDL-C levels require supplementary treatment. Within a particular investigation, it was shown that the percentage of patients who were able to achieve an LDL-C value below 55 mg/dL was as low as 18.7%. This study focused on a population where more than half of the individuals regularly consumed high-intensity statin medication [25]. Therefore, it is recommended to combine additional medicines such as ezetimibe and PCSK9 inhibitors (PCSK9-I) with the highest tolerated statin in order to further decrease LDL-C levels toward the desired target [22]. In addition to reducing cholesterol levels, lipid-lowering medications have several pleiotropic effects that could improve the clinical outcomes of individuals who have experienced acute coronary syndrome (ACS). One of the multiple effects of statins, for example, is the stabilization of plaques by increasing fibrous cap thickness and reducing microcalcification. Statins also have a plaque regression effect by removing lipid and necrotic core, restoring endothelial function, and ceasing the proliferation of smooth muscle cells within blood vessels [26].

Presently, the guidelines favor the use of PCSK9-I, a monoclonal antibody that specifically targets PCSK9, in conjunction with statin-based therapy for the management of dyslipidemia [22]. In humans, PCSK9 regulates the recycling of LDL receptors (LDL-R), with inhibition of PCSK9 leading to increased LDL receptors present at the cell surface for binding and removal of circulating LDL particles [27]. Prior research has demonstrated the efficacy of PCSK9 inhibitors in preventing atherosclerotic events by significantly lowering LDL cholesterol levels, similar to statins. Moreover, these medications might be deemed as safe and well-tolerated. Nevertheless, there are still some disputes over the effectiveness of these medications in lowering mortality, as well as a lack of data on their pleiotropic effects, such as their impact on the characteristics of atherosclerotic plaque, and their long-term safety [28]. This systematic review and meta-analysis describe that addition of PCSK9 on statin-treated patients with ACS is correlated with a significant amelioration in safety and efficacy profile and is supported by the result of lipid plaque phenotype changes. This study found a significant amelioration in all lipid profiles that were mentioned thus it is postulated that these findings will be beneficial to the patients. The effect is supported by the significant change of plaque phenotype in terms of TAV, PAV, FCT, LA, QFR, and DoS but not in lipid/lesion length. This study also did not find any significant differences in alirocumab and evolocumab thus proving that both PCSK9 inhibitors have the same potencies. The sub-analysis of the ODYSSEY trials included a qualitative analysis that supports the findings. It states that out of 17,589 patients with LDL-C levels above 54 mg/dL, despite receiving optimized statin therapy and not taking ezetimibe, the addition of alirocumab enabled the majority of patients (94.6%) to achieve the LDL-C goal of less than 54 mg/dL [19].

In this meta-analysis, the authors classify events such as mortality and re-infarction in the safety analysis. Based on the safety analysis, there is a general reduction in the occurrence of adverse events, particularly in severe cases like death and myocardial infarction. However, there is no decrease in less severe adverse effects such as injection site reactions, myalgia, pain, and other non-emergency and significant events. Previous studies have provided support for the correlation between lipid profile, plaque phenotype, and the occurrence of serious adverse effects. These studies have found that the presence of lipid-rich plaque in the evaluated target vessels, as determined by OCT, is indicative of an increased risk for future major adverse cardiac events (MACE), including cardiac death and re-infarction. Additionally, a recent meta-regression analysis has shown that improvements in the lipid plaque phenotype are associated with a decrease in the likelihood of MACE [29, 30]. The consequences of plaque regression in the result of safety and efficacy in patients with ACS can be explained by one of the mechanisms of ACS, namely plaque rupture with systemic inflammation [31]. In the context of ACS, inflammation plays a crucial role as the primary regulator of the fragility of the fibrous cap. To prevent the events, such as plaque rupture, it is possible to reduce the lipid content and increase the thickness of the fibrous cap in the plaque by including PCSK9 inhibitors in the therapy regimens. Additionally, PCSK9 inhibitors can suppress the inflammatory pathway, which is known to be activated by PCSK9 and leads to the expression of pro-inflammatory genes through the activation of nuclear factor kappa beta (Nf-kB). However, it should be noted that the analysis of inflammation is not within the scope of this meta-analysis [32]. It is important to emphasize that these conclusions remained consistent with previous studies thus supporting the validity of this meta-analysis [6, 7].

This meta-analysis is consistent with previous meta-analysis by Wu et al*.* which examined the effect of combining PCSK9 inhibitors with statin therapy on atherosclerotic plaque regression compared to statin therapy alone [33]. Research findings indicate that the combination of PCSK9 inhibitors and statin therapy led to a notable reduction in the percent atheroma volume (PAV), total atheroma volume (TAV), and lipid arc. Additionally, there was an increase in fibrous cap thickness (FCT) of the coronary atherosclerotic plaque. However, these effects were observed in patients with coronary artery disease (CAD) and not specifically in patients with acute coronary syndrome (ACS). However, several of the studies included in Wu et al.'s meta-analysis were not double-blinded randomized controlled trials (RCTs) or were not RCTs at all, which could potentially impact the treatment outcomes. This meta-analysis comprehensively evaluated the safety and effectiveness of PCSK9 inhibitors in patients who were already receiving statin treatment, with a specific focus on the use of plaque phenotyping data in patients with acute coronary syndrome (ACS). Therefore, minimizing the likelihood of prejudice. The studies included in the analysis were evaluated both quantitatively and qualitatively across many characteristics, including plaque phenotype, changes in lipid profile outcomes, and adverse events associated with medication.

Our meta-analysis study serves to reinforce the findings of the prior meta-analysis carried out by Ahmed Atia et. al., which demonstrates that the use of PCSK9 inhibitors as a secondary prevention medication in patients following ACS can significantly enhance lipid profiles and decrease the occurrence of cardiac events [24]. Furthermore, our findings in this publication demonstrate that the combination of PCSK9 inhibitors and statins in secondary prevention approach can alter morphology and successfully impede the advancement of atheroma plaque formation. Eventually, the findings from these two meta-analyses demonstrate the criticality of secondary prevention in patients who have experienced acute coronary syndrome/coronary artery disease. The importance of secondary prevention in this population requires that patients with cardiovascular disease must take statins for the lifetime. This is a concern in its own right, particularly regarding patient compliance/adherence with statin administration. The findings of a cross-sectional study conducted by Tuba Ozdemir et. al. revealed that 46.7% of the 300 patients were categorized as non-adherence [34].A majority of non-adherence patients, specifically 60%, were caused by physician discontinuation of the statin prescription and 8% of patients received negative information from TV programs and social media [34]. This will further burden the patient as statin-only treatment is proven to be ineffective in some patients and needs to be combined with another medication such as PCSK9 inhibitors. To overcome this problem, a finding by meta-analysis carried out by Faysal Saylik et. al., showed a substantial decrease of 63% in the non-adherence rate to statins among patients who implemented digital health [35]. Digital health interventions might provide improved outcomes for patient care by providing closer follow-up, compared to standard care. Utilizing a digital health platform like mobile-health (m-health) through devices plays a crucial role in enhancing the doctor-patient interaction, ensuring effective follow-up and maintaining the consistency of prescriptions, a fundamental aspect for adherence [35, 36]. Utilizing digital health platform would also becoming a better approach to enhance the implementation of PCSK9 inhibitors in addition of statin-only treatment. In other words, enhancing patient adherence will yield significant advantages and secondary preventive effects of statins or PCSK9 inhibitors in this meta-analysis study can be effectively applied.

The main limitation of this study was the heterogeneity of the individual studies. Limitation was present in our meta-analysis which affected in clinical heterogeneity because of differences in the therapeutic regimen that were used such as dosages and administration intervals, and the heterogeneity of adverse effects on included studies. Furthermore, some studies had a small number of participants taking ezetimibe, which resulted in a lack of uniformity in the baseline characteristics of the patients, although this did not have a significant impact. Despite the moderate to high level of heterogeneity, the Randomized Controlled Trials (RCTs) and Cohorts included in this systematic review and meta-analysis were of good quality, as determined by the Modified Jadad Scale and Newcastle–Ottawa Scale (NOS) assessment. On the other hand, the findings of this study are able to encourage the cardiology societies of any nation to force the National Health Regulator to cover or provide PCSK9 inhibitors in order to enhance the secondary prevention efforts for patients after experiencing ACS or any cardiac event.

## Conclusion

In the event PCSK9 inhibitors were added to statin treatment, there were predominantly significant positive effects in terms of safety and effectiveness. This was corroborated by results showing a reduction in coronary plaque in patients who had recently experienced an acute coronary syndrome (ACS). Additional research is required to investigate the effects of PCSK9 inhibitors with consistent dosage, medication type, and therapy duration, specifically in individuals who are solely treated with statins.

## Supplementary Information

Additional file 1

## Data Availability

The data underlying this article are available in the article and its supplementary materials.

## Abbreviations

ACS: Acute coronary syndrome
Apo B: Apolipoprotein B
ASCVD: Atherosclerotic cardiovascular disease
CAD: Coronary artery disease
CI: Confidence interval
cTn: Cardiac troponin
DALYs: Disability-adjusted life-years
DoS: Diameter of Stenosis
ECG: Electrocardiogram
FCT: Fibrous cap thickness
HDL-C: High density lipoprotein cholesterol
IVUS: Intravenous ultrasonography
LA: Lipid Arc
LCBI: Lipid core burden index
LDL-C: Low density lipoprotein cholesterol
LDL-R: Low density lipoprotein receptor
MACE: Major cardiac adverse events
MD: Mean difference
Nf-kB: Nuclear factor kappa beta
NOS: Newcastle–Ottawa Scale
NSTEMI: Non-ST elevation myocardial infarction
OCT: Optical coherence tomography
PAV: Percent atheroma volume
PCSK9: Proprotein convertase subtilisin/kexin type 9
PICO: Patient, Intervention, Comparison, Outcome
PRISMA: Preferred Reporting Items for Systematic Reviews and Meta-analysis
PROSPERO: Prospective Register of Systematic Reviews
QCA: Quantitative coronary angiography
QFR: Quantitative flow ration
RCT: Randomized controlled trial
RR: Risk ratio
SD: Standard deviation
STEMI: ST elevation myocardial infarction
SMD: Standardized mean difference
TAV: Total atheroma volume
TC: Total cholesterol
TG: Triglycerides
